# Mechanochemical Solid-State Immobilization of Photofunctional Dyes on Amorphous Silica Particles and Investigation of Their Interactive Mechanisms

**DOI:** 10.3390/nano14090741

**Published:** 2024-04-24

**Authors:** Reo Kimura, Sunao Chatani, Masahiko Inui, Satoshi Motozuka, Iori Yamada, Motohiro Tagaya

**Affiliations:** 1 Department of Materials Science and Bioengineering Technology, Nagaoka University of Technology, Kamitomioka 1603-1, Nagaoka 940-2188, Japan; s181027@stn.nagaokaut.ac.jp (R.K.); iori_yamada@vos.nagaokaut.ac.jp (I.Y.); 2 Production Engineering Department, Ohara Quartz, Minato 1850, Wakayama 640-8404, Japan; 3 Department of Materials Science and Engineering, Kyushu Institute of Technology, Sensuicho 1-1, Tobata-ku, Kitakyushu 804-8550, Japan; motozuka@post.matsc.kyutech.ac.jp

**Keywords:** amorphous silica particles, mechanochemical reaction, Cl-containing ASPs, photofunctional dyes, fluorescein, methylene blue, immobilization techniques

## Abstract

Amorphous silica particles (ASPs) have been reported to exhibit bioactive properties and are becoming the focus of attention as bioceramics. However, their interactions with proteins in living organisms remain to be understood and need to be investigated in order to achieve wider applications. Our research group found that chlorine (Cl)-containing ASPs are useful for protein immobilization. Photofunctional dyes (fluorescein (FS^−^), methylene blue (MB^+^)) that have the carboxy and amino groups as the main functional groups were immobilized on the Cl-containing ASPs via the mechanochemical method as the model molecule and their spectral properties were used to investigate and discuss the organic/inorganic interfacial bonding states. In FS^−^, the oxygen atoms of the carboxy groups in the molecule were immobilized by the hydrogen bonds with the silanol groups on the ASPs surfaces, indicating that there is an optimum Cl content for the immobilization as the monomer state. In the case of MB^+^, as the Cl concentration in the ASPs increases, the immobilization via the electrostatic interactions between the Cl in the ASPs and the terminal dimethylamino group, and the hydrogen bonding between the N atoms of the MB^+^ hetero ring and the particle silanol group were enhanced. These results mainly suggest that the protein adsorption system occurs through the hydrogen bonding between the carboxy groups of the protein and the silanol groups on the particles and via electrostatic interactions between the amino groups of the protein and the dissociated silanol groups and the contained Cl at the particles. Thus, the spectral characterization using dyes as probes is expected to predict the protein interactions with the amorphous silica particles.

## 1. Introduction

Amorphous silica particles (ASPs, SiO_2_) are useful as biomaterials [1,2,3,4,5]. Therefore, ASPs have been used for drug delivery carriers based on protein immobilization and for supplements and cosmetics [6,7,8]. However, in the applications, the basic interactions between biological molecules and ASPs at the interface level have not yet been investigated. Generally, there are electrostatic interactions, hydrogen bonding, and hydrophobic interactions in biomolecular immobilization [9,10,11]. Our research group has also investigated the adsorption state of the antibody proteins on the Cl-containing ASPs while maintaining their highly ordered protein structures [12]. However, the functional groups in the protein components are very complex, and their interactions with the ASPs (≡Si−OH, ≡Si−O−Si≡, ≡Si−Cl, etc.) are difficult to predict and are desired to be clarified in the nanomaterial chemistry field. Therefore, the adsorption behavior of the proteins can be precisely controlled by investigating the interactive functional groups of the proteins, leading to a wider range of biomedical applications of ASPs.

To investigate the interactive functional groups, the adsorption of photofunctional dyes on silica surfaces was applied to this study. In particular, fluorescein (FS^−^) and methylene blue (MB^+^) can clarify the surface properties of silica-based materials through their fluorescence and absorption spectra [13,14,15]. These dyes contain the main functional groups for the proteins (carboxy groups, amino groups, etc.) [16,17] and their adsorption states could predict the interactions of biomolecules with ASPs. So far, the adsorptive immobilization of FS^−^ and MB^+^ molecules from the liquid phase on the silane coupling agent-modified ASPs has been studied extensively [18,19,20], and the direct interfacial bonds between biomolecules and silica have not been understood.

Mechanochemical solid-state reactions, which provide the mechanical energy to the objective materials to resultantly activate the surfaces and to form the new chemical bonds without solvation, have been used to immobilize the dyes on the inorganic solids. The methods are very useful in terms of direct and effective interfacial reactions [21,22,23]. Thus, the mechanochemical immobilization of FS^−^ and MB^+^ on inactive ASPs will be achieved. In practice, the mechanochemical immobilization of dyes on the inorganic solids (clay, apatite, etc.) has been applied to identify the exposed atoms as well as immobilization [24,25,26]. These studies focused on their molecular photofunctional performances on confined nanospaces or nanomaterials, which are mainly applied to optoelectronic devices or bioimaging applications. In this study, the interactions of ASPs with functional groups for protein immobilization were investigated using the photospectral properties of the photofunctional dyes containing amino and carboxy groups.

**Scheme 1:**
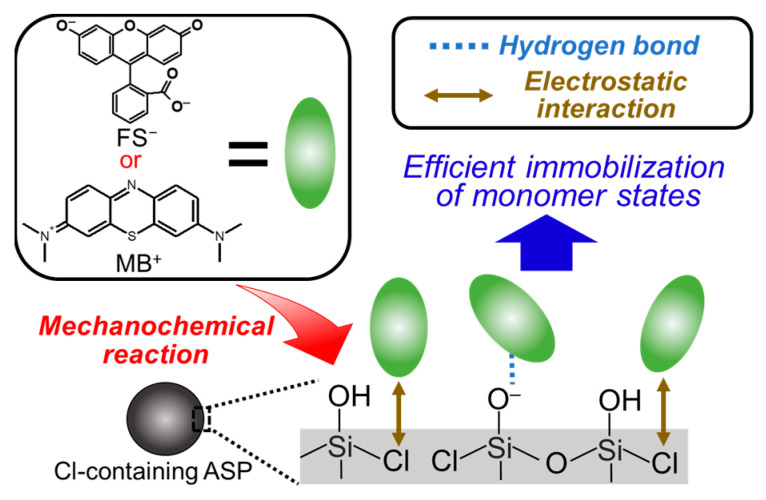
Illustration of the mechanochemical immobilization of the photofunctional dyes on the Cl-containing ASPs in this study.

In this study, ASPs or chlorine-containing ASPs were reacted with FS^−^ and MB^+^ via the mechanochemical method, and the photospectral properties were evaluated for the clarification of the interactions between the dye functional groups and ASPs (Scheme 1), leading to effective interactions between biomolecules and ASPs for biomedical applications. This study will achieve the supplementary prediction of the immobilization states of the proteins using simplified photofunctional dyes with carboxy and amino groups as the prelude to the complete prediction in the future. Particularly, the proteins have a multifunctional and complex structure, and their immobilization states are determined by many factors, such as the environment surrounding the protein, including the influence of water molecules and ions, which prevents the prediction of interactions between the protein and ASPs. Therefore, as the prelude to the complete prediction of the immobilization states of the proteins, we aimed to achieve the prediction using simplified photofunctional dyes with carboxy and amino groups.

## 2. Materials and Methods

### 2.1. Synthesis of Cl-Containing ASPs

ASPs and Cl-containing ASPs were synthesized via the Vapor-Phase Axial Deposition method as previously reported by our previous paper [12]. The chlorine-free particles were called ASPs. The particles containing the lower and higher concentrations of chlorine were called L-CL and H-CL. All the particles exhibited amorphous and spherical particles, and the average particle diameters of ASPs, L-CL, and H-CL were 139, 135, and 143 nm, respectively. The particles were non-porous, and the chlorine concentrations of L-CL and H-CL were 0.05 and 0.40 mol%. The surface silanol group fraction of the particles, which was measured via XPS, of ASPs, L-CL, and H-CL were 15, 60, and 85%, indicating that the silanol group density increased with the increasing chlorine concentration.

### 2.2. Mechanochemical Immobilization of Photofunctional Dyes

FS^−^ (purity: <100%, product no.: 065-00252) and MB^+^ (purity: 98.5+%, product no.: 133-06962) were purchased from FUJIFILM Wako Pure Chemical Corp., Osaka, Japan. The synthesized particles and photofunctional dyes (FS^−^ or MB^+^) were mixed in an agate mortar (mortar diameter and depth: 6.6 and 3.8 cm; rod length and diameter: 8.3 and 2.5 cm) at the weight ratio of 5:1 for the mixing time of 10 min at room temperature. The resulting powder mixture of the particles and dyes was washed with either EtOH, IPA, or ultrapure water (FS^−^ only), and the solid phase was obtained via centrifugation (3 °C, 13,000× *g*, 10 min). The washing process was repeated until the supernatant solution became clear. However, it was difficult to achieve clarity by washing only with ultrapure water. The washed particles were dried overnight at 60 °C to obtain ASPs and Cl-containing ASPs immobilized with FS^−^ and MB^+^. FS^−^ and MB^+^ in the solution were also prepared for the comparison. In particular, both FS^−^ and MB^+^ powders were dissolved in 1 mM of phosphate buffer at the concentration of 1.0 × 10^−7^ M. Here, the buffer was prepared by mixing sodium dihydrogen phosphate (purity: 99+%, product no.: 197-02865) with sodium hydrogen phosphate (purity: 99+%, product no.: 197-09705), which were purchased from FUJIFILM Wako Pure Chemical Corp., Osaka, Japan.

### 2.3. Characterization of the Mechanochemically Treated ASPs

The crystalline structures and photospectral properties of the particles were characterized with X-ray diffraction (XRD: Smart Lab, Rigaku Co., Ltd., Tokyo, Japan), UV-Vis absorption spectrometry (UV-Vis: JASCO Corp., Tokyo, Japan, V-750) with an integrating sphere (ISV-922, JASCO Co., Ltd., Tokyo, Japan), fluorescence microscopy (FL: λ_ex_ = 465–495 nm, λ_em_ = 515–555 nm, exposure time = 100 ms), and photoluminescence spectrometry (PL: JASCO Corp., Tokyo, Japan, FP8500).

The structural characterization was evaluated with XRD using the CuKα line (λ: 1.5418 Å) as the X-ray source with a voltage/current of 40 kV/30 mA and a scan speed of 3.0°/min.

**Scheme 2:**
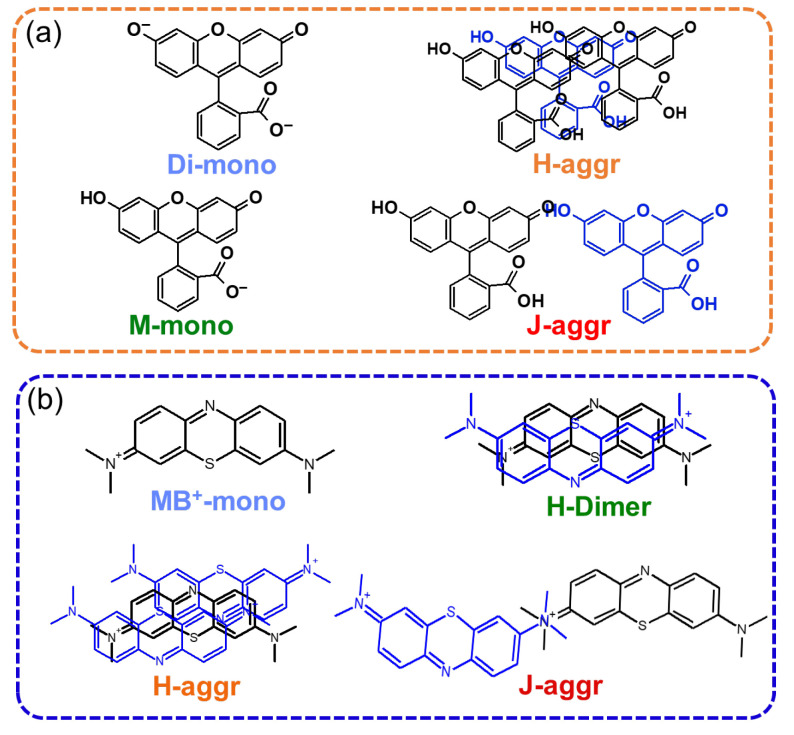
Illustration of the aggregation structures among (**a**) FS^−^ and (**b**) MB^+^.

To investigate the immobilization molecular state of FS^−^ and MB^+^ on the particles, PL spectrophotometry for FS^−^ and diffuse reflectance absorption spectrophotometry for MB^+^ were used in this study. For FS^−^, the PL spectra were recorded under the excitation wavelengths of 440–450 nm in the PL range of 480–600 nm. Spectral peak separation and deconvolution were conducted for the evaluation. In particular, FS^−^ and MB^+^ show the molecular aggregation states as shown in Scheme 2. FS^−^ can take the aggregation structures of monomeric monoanion (M-mono), J-aggregate (J-aggr), monomeric anion (Di-mono), and H-aggregate (H-aggr) (Scheme 2a), and MB^+^ can take the aggregation structures of J-aggregate (J-aggr), monomer (MB^+^- mono), H-dimer (H-dimer), and H-aggregate (H-aggr) forms (Scheme 2b). Based on the above molecular aggregation states, the FS^−^ and MB^+^ immobilized on the particles were determined separately and deconvoluted with the four components on the basis of previous reports (Table 1) [13,14,15]. All the deconvolutions were performed by fitting with the Gaussian function conducted through the Experimental Procedure S1. Then, the area of the spectrum of each component was divided by the total area of the measured spectra to determine the proportions (%).

## 3. Results and Discussion

The XRD patterns of the FS^−^-immobilized ASPs are shown in Figure S1. In all the amorphous halo diffractions, there were no differences in the Cl concentration. In the FS^−^-immobilized ASPs washed with EtOH and IPA (Figure S1a,b), there was no diffraction due to the crystallized FS^−^, and those washed with ultrapure water showed diffraction due to the crystalline FS^−^, indicating the residual FS^−^ molecules with the aggregation states.

The photographs of the FS^−^-immobilized ASPs are shown in the Figure S2 inset. The powders washed with EtOH and IPA (Figure S2a,b) exhibited a light-yellow coloration, and those washed with ultrapure water (Figure S2c) showed an orange coloration as in the case of the FS^−^ powder alone, indicating the residual aggregation states of FS^−^ only when washed with ultrapure water. The diffuse reflectance absorption spectra of the FS^−^-immobilized ASPs washed with EtOH and IPA (Figure S2a,b) exhibited the absorption bands due to the FS^−^ monoanion and dianion monomer states [27]. In the FS^−^-immobilized ASPs washed with ultrapure water, the absorption band peak due to the aggregation states has a higher intensity, which also showed the aggregation states of FS^−^.

The excitation spectra of the FS^−^-immobilized ASPs washed with EtOH and IPA (Figure S3a–d) showed the excitation peaks due to the monoanion and dianion monomers of FS^−^, consequently providing the green-colored fluorescence. The fluorescence microscopy images of the FS^−^-immobilized ASPs washed with EtOH and IPA (Figure S3g,h) also showed a green color. In the particles washed with ultrapure water (Figure S3e,f), the excitation and fluorescence spectral shapes were almost the same as in the case of those washed with EtOH and IPA, and the fluorescence intensity was much lower. It was suggested that the π-stacking states among the FS^−^ molecules would remain as the aggregation states. According to the previous report for the FS powder alone [15], the fluorescence spectra could not be observed, indicating the occurrence of fluorescence quenching in the present study.

The molecular aggregation states of FS^−^ in solution were measured and investigated through the deconvolution and separation of the fluorescence spectra (Figure S4). Although the fitting accuracy was not good, the majority of the M-mono fraction was observed.

The FS^−^ molecular aggregation states on the ASPs washed with ultrapure water were determined via the spectral separation technique for the fluorescence spectra (Figure S5). For the molecular aggregation state of FS^−^, the fraction of Di-mono of FS^−^ was lower and those of J-aggr and H-aggr were higher (Figure S5b), leading to the lower fluorescence intensity with the π–π molecular stacking of FS^−^.

**Figure 1:**
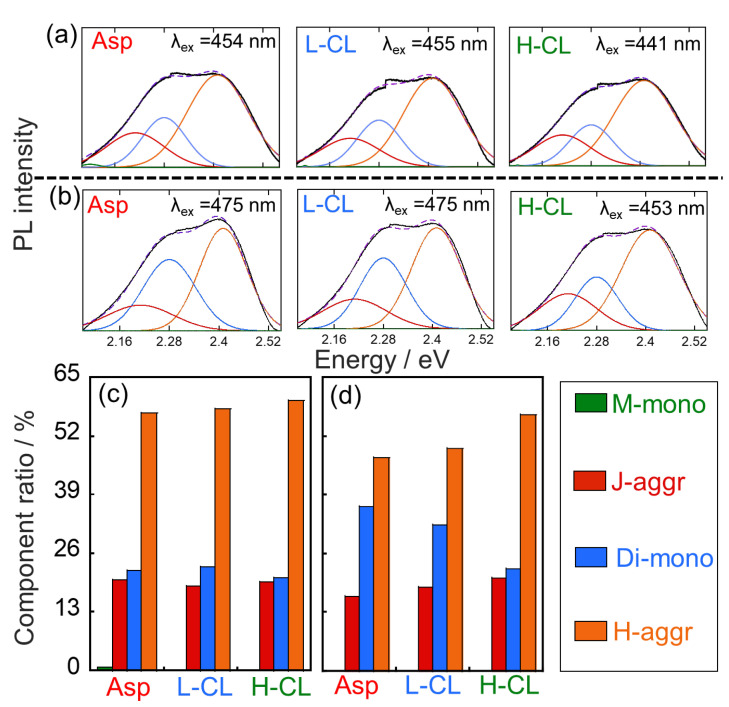
(**a**,**b**) Luminescence spectral separation results for four aggregate states and (**c**,**d**) the component ratios for four aggregate states calculated with the spectra of the FS^−^-immobilized ASPs washed with (**a**,**c**) EtOH and (**b**,**d**) IPA.

The molecular aggregation states of the FS^−^ on the particles washed with EtOH and IPA were characterized via the fluorescence spectra’s spectral separation technique (Figure 1). The fluorescence intensities were sufficiently detected to enable the fitting (Figure 1a,b). As compared with the other cases, the higher fraction of FS^−^ Di-mono state was clearly detected. For the FS^−^ molecular aggregation states washed with EtOH (Figure 1c), no change in the fraction of Di-mono and the increase in the H-aggr fraction were observed with increasing the Cl concentration. In the washed-with-IPA case (Figure 1d), the decrease in the Di-mono fraction and the increase in the J-aggr and H-aggr fractions were significantly observed with the increasing Cl concentration. The fraction of M-mono in all the ASPs was lower. From these results, these molecular states would be attributed to the different surface states of the particles. In detail, the Cl concentration destabilized the siloxane bonds in the silica network, resulting in increased silanol groups at the topmost surfaces. Accordingly, the number of reactive sites where the hydrogen bonds can be formed between the oxygen atoms of the carboxy groups of the FS^−^ and ≡Si−OH of the ASPs increased with the increasing Cl concentration. Therefore, there is the fraction of the optimum silanol groups for the immobilization of FS^−^ at the solid-state reaction, and the excess Cl concentration would increase the FS^−^ molecular aggregation states on the ASPs, indicating the importance of the optimum silanol group fraction for the immobilization of Di-mono. In fact, it has been reported that the optimum FS^−^ concentration in solution with the higher fraction of Di-mono without forming the aggregates is less than 1.0 × 10^−6^ M [28,29], indicating the optimum Cl concentration (i.e., silanol group fraction) as well as the effective washing solvent for the solid-state reaction between ASPs and FS^−^.

The XRD patterns of the MB^+^-immobilized ASPs are shown in Figure S6. All the samples had amorphous halo diffractions, and there was no change in the Cl concentration. In all the MB^+^-immobilized ASPs (Figure S6a,b), there was no diffraction due to the crystallized MB^+^.

**Figure 2:**
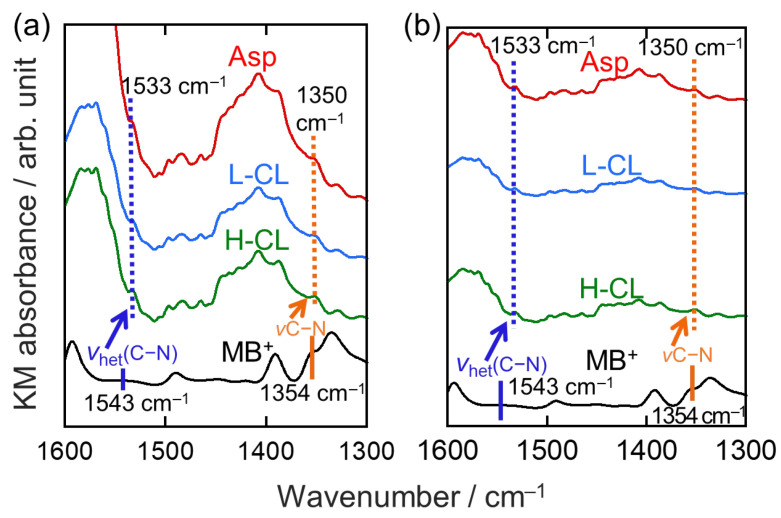
FT-IR spectra of the particles washed with (**a**) EtOH and (**b**) IPA, and the MB^+^ powder alone, which shows the C–N absorption bands.

The chemical bonding states of the MB^+^-immobilized ASPs were measured with the FT-IR spectra (Figure 2). In the MB^+^-immobilized ASPs washed with EtOH and IPA (Figure 2a,b), the C–N stretching vibrations due to the heterocycle and due to the terminal dimethylamino group of MB^+^ were significantly red-shifted as compared with the case in the MB^+^ powder alone treated with the mechanochemical method. It is suggested that the terminal dimethylamino group is positively charged, which bonds to the silanol group and Cl ion in ASPs with electrostatic interactions. Moreover, the N atom in the hetero ring of MB^+^ would interact with the silanol groups via hydrogen bonding [30,31].

The diffuse reflectance absorption spectra of the MB^+^-immobilized ASPs were measured (Figure S7). All the ASPs exhibited a light-blue color (Figure S7, inset). There were no differences between the absorption spectra of the ASPs washed with EtOH and IPA (Figure S7a,b), which were similar to the spectral shape of the MB^+^ in solution.

The molecular aggregation states of MB^+^ in solution were measured and investigated via the deconvolution and separation of the UV-Vis absorption spectra (Figure S8). The MB^+^-mono and H-Dimer fractions were dominantly observed.

**Figure 3:**
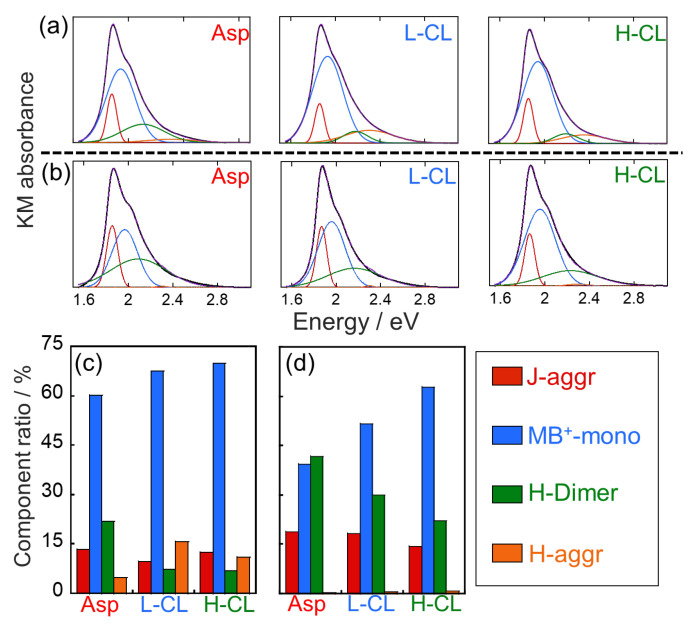
(**a**,**b**) UV-Vis diffuse reflectance absorption spectral separation results for four aggregate states and (**c**,**d**) component ratios of the MB^+^-immobilized ASPs washed with (**a**,**c**) EtOH and (**b**,**d**) IPA.

The molecular aggregation states of the MB^+^ on the ASPs washed with EtOH and IPA were characterized via the spectral separation technique for the diffuse reflectance absorption spectra (Figure 3a,b). In all the samples, the residual values by the deconvolutions were less than 0.22%, indicating that the fitting accuracy was good. For the molecular aggregation states of the MB^+^ in the ASPs washed with EtOH (Figure 3c), the fraction of MB^+^-mono increased with the increasing Cl concentration, and the decrease in the H-Dimer fraction was simultaneously observed. In the molecular aggregation states of MB^+^ in the ASPs washed with IPA (Figure 3d), the fraction of MB^+^-mono was lower than in the case of the ASPs washed with EtOH. With the increasing Cl concentration, the increase in the fraction of MB^+^-mono and the decrease in the J-aggr and H-Dimer were significantly observed. In the ASPs, the fraction of the aggregation is relatively larger because of the smaller number of silanol groups on the topmost surface. Accordingly, the N atom of the hetero ring in the MB^+^ interacted with the silanol groups in the ASPs, dominantly showing hydrogen bonding, and the adsorption sites are smaller in number, resulting in the formation of the aggregation form on the MB^+^-immobilized ASPs. In the MB^+^-immobilized Cl-containing ASPs, the fraction of the terminal dimethylamino group bound to the Cl and ≡Si−O^−^ in the L-CL and H-CL with electrostatic interactions increased with the increasing Cl concentration [12], indicating the effective immobilization of the MB^+^-mono state. In the adsorptive immobilization of MB^+^ from the liquid phase on ASPs, it has been reported that the MB^+^-dimer was effectively formed [32], and understanding the interfacial interactions was difficult, suggesting that the mechanochemical solid-state reactions are suitable for the monomeric immobilization to predict the simple molecular interactions with ASPs.

**Scheme 3:**
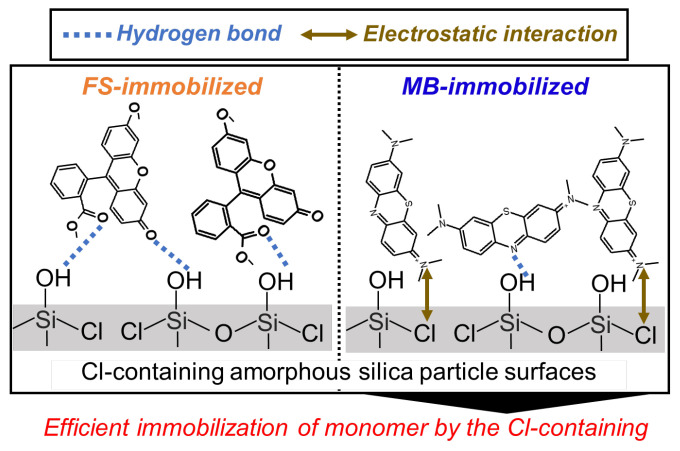
Illustration of the immobilization states of the photofunctional dyes on the Cl-containing ASP surfaces.

From the results, the adsorption states of the photofunctional dye molecules on the Cl-containing ASPs are shown in Scheme 3. In the FS^−^ system, the O atoms of the carboxy groups could be immobilized to the silanol groups on the ASPs via hydrogen bonding. Thus, acidic proteins such as albumin and streptavidin, etc. [33,34], which are negatively charged in biological fluid, can be immobilized via the carboxy group to the silanol group via hydrogen bond. In the MB^+^ system, the terminal dimethylamino group of MB^+^ was immobilized to the Cl and ≡Si−O^−^ in the ASPs through electrostatic interactions, and the N atom of the heterocyclic ring in MB^+^ was also immobilized to ≡Si−OH in the ASPs via hydrogen bonding. Therefore, the positively charged basic proteins in biological fluid [35] could be immobilized on the Cl-containing ASPs through electrostatic interactions at the N-terminal and hydrogen bonding at the C-terminal.

## 4. Conclusions

The photofunctional dyes, which have the carboxy and amino groups as the model molecules, were immobilized on the Cl-containing ASPs via a mechanochemical method. The spectroscopic properties were used to investigate and discuss the interfacial dye–silica binding states. In FS^−^, the O atom of the carboxy group could be immobilized on the silanol groups of the ASPs with hydrogen bonding. In MB^+^, there were two types of immobilization states, which are via the electrostatic interaction between the Cl^−^ or ≡Si−O^−^ in the ASPs and the terminal dimethylamino group, and via the hydrogen bonding between the N atom of the MB^+^ hetero ring and the silanol group of the Cl-containing ASPs. It was suggested that the carboxy group of the protein interact with the silanol groups on the particles through hydrogen bonding and that the amino group of the protein interact electrostatically with the Cl^−^ or ≡Si−O^−^ in the ASPs. We have already demonstrated that the containing of Cl in ASPs induces the generation of ≡Si–Cl bonds as well as the increased density of ≡Si−O^−^ on the surfaces [12], leading to effective electrostatic immobilization as monomer states. In particular, the surface chemical bonding states of the Cl-containing ASPs measured with XPS spectra (i.e., the narrow scan deconvolution of the Si(2*p*) and O(1*s*) spectra) demonstrated that the proportion of Si–OH increased with the increasing Cl-containing concentration, which would be due to the cleavage links in the silica networks through the ≡Si–Cl bond formation. In fact, the inclusion of Cl in ASPs significantly increased the ≡Si–Cl bonding and Si–OH surface functional groups of the particles. Thus, the presence of Cl was expected to efficiently improve surface reactivity. Moreover, via the FT-IR spectra, the (≡Si–Cl···HO–) absorbance peak significantly increased with the increasing Cl doping concentration. Thus, the ≡Si–Cl in the top surface layer of L-CL and H-CL interacted with the OH groups through weak hydrogen bonding, which is expected to contribute to the mild surface reactivity of the Cl-containing ASPs. Therefore, spectral characterization using the photofunctional dyes as the probes would be expected to predict the interactions with the surfaces of ASPs. It leads to the mechanochemical solid-state hybridization techniques between organic and inorganic materials as well as the precise control of the immobilization states of proteins on solid surfaces. Furthermore, this is the first prediction method for the direct interactions between the surfaces of ASPs and the functional groups of the proteins using photofunctional dyes, which will also be applicable for investigating the surface properties of ASPs.

## Figures and Tables

**Table 1:** Peak assignment of four-component aggregation states of the FS^−^-immobilized ASPs, FS^−^ solution, MB^+^-immobilized ASPs, and MB^+^ solution, respectively, in the luminescence (FS^−^) and UV-Vis diffuse reflectance absorption spectra (MB^+^). The different colored font indicates the variation of the dye molecular aggregation, which correspond to the separated spectral colors.

	Assignments	Peak Position [nm]	Peak Position [eV]
FS^−^-immobilized ASPs	M-mono	590	2.1
	J-aggr	561	2.21
	Di-mono	544	2.28
	H-aggr	515	2.41
FS^−^ solution	M-mono	564	2.2
	Di-mono	535	2.32
	H-aggr	514	2.41
MB^+^-immobilized ASPs	J-aggr	708–664	1.75–1.87
	Di-mono	644–607	1.93–2.04
	H-Dimer	592–548	2.09–2.26
	H-aggr	539–518	2.30–2.39
MB^+^ solution	J-aggr	761	1.63
	Di-mono	663	1.87
	H-Dimer	608	2.04
	H-aggr	577	2.15

## Data Availability

The data are contained within the article.

## Supplementary Materials

The following supporting information can be downloaded at https://www.mdpi.com/article/10.3390/nano14090741/s1: Figure S1: XRD patterns of the FS^−^-immobilized ASPs washed with EtOH, IPA, and ultrapure water; Figure S2: UV-Vis diffuse reflectance absorption spectra of the FS^−^-immobilized ASPs washed with EtOH, IPA, and ultrapure water (inset: photographs of the powder states); Figure S3: Excitation and luminescence spectra (EtOH-, IPA-, and ultrapure water-washed) of the FS^−^-immobilized ASPs. The monitored luminescence and excitation wavelengths for the spectra are represented by λ_em_ and λ_ex_. Fluorescent microscope images of the FS^−^-immobilized ASPs washed with the EtOH and IPA (λ_ex_ = 465−495 nm, λ_em_ = 515−555 nm, exposure time = 100 ms); Figure S4: Luminescence spectral separation result for four aggregate states of the FS^−^ solution (phosphate buffer (1 mM), pH = 7.4), and the detailed assignments are shown in Table 1. Component ratio for four aggregate states was calculated with the separated spectra; Figure S5: Luminescence spectral separation results for four aggregate states of the FS^−^-immobilized ASPs washed with the ultrapure water, and the detailed assignments are shown in Table 1. Component ratio for four aggregate states was calculated via the separated luminescence spectra; Figure S6: XRD patterns of the MB^+^-immobilized ASPs washed with the EtOH and IPA; Figure S7: UV-Vis diffuse reflectance absorption spectra of the MB^+^-immobilized ASPs washed with EtOH and IPA (inset: photographs of the powder states); Figure S8: UV-Vis absorption spectral separation result for four aggregate states of the MB^+^ solution (phosphate buffer (1 mM), pH = 7.4), and the detailed assignments are shown in Table 1. Component ratio for the four aggregate states.
