# Supplementary material for: Mechanochemical Solid-State Immobilization of Photofunctional Dyes on Amorphous Silica Particles and Investigation of Their Interactive Mechanisms
Source: Nanomaterials (Basel). 2024 Apr 24;14(9):741. doi: 10.3390/nano14090741 (PMC11085052; doi:10.3390/nano14090741)
Supplement: Supplementary file 1 [file nanomaterials-14-00741-s001.zip › nanomaterials-2946871-supplementary.pdf]

Supplementary Materials

Mechanochemical solid-state immobilization of photofunctional dyes on amorphous silica particles and investigation of their interactive mechanism

Reo Kimura, [1] **Sunao Chatani,** [2] **Masahiko Inui,** [2]

Satoshi Motozuka, [3] **Iori Yamada,** [1] **Motohiro Tagaya** [1,*]

[1] *Department of Materials Science and Bioengineering Technology, Nagaoka University of Technology, Kamitomioka 1603-1, Nagaoka 940-2188, Japan*

[2] *Production Department, Ohara Quartz, Minato 1850, Wakayama, Wakayama 640-8404, Japan*

[3] *Department of Materials Science and Engineering, Kyushu Institute of Technology, Sensuicho 1-1, Tobata-ku, Kitakyushu 804-8550, Japan*

Author to whom correspondence should be addressed:

* Tel: +81-258-47-9345, Fax: +81-258-47-9300, E-mail: tagaya@mst.nagaokaut.ac.jp

Experimental Procedure S1

All the deconvolutions were performed by fitting with the Gaussian function (**Eq. S1)**). Here, *A* was the height of the peak, *B* was the position of the peak, *C* was the half-width.

$$f(x) = Aexp\left(\frac{(x-B)^2}{C^2}\right) \quad \textbf{(Eq. S1)}$$

For the fitting, the square of the difference between the measured value (y_i) at each wavenumber position (x_i) and the sum of each component ($F(x_i)$) were calculated **(Eq. S2)**, and the residual sum of squares (*RSS*) **(Eq. S3)** were obtained by the summing the squared differences over the fitting region and minimized to the minimum value. The Generalized Reduced Gradient (GRG) nonlinear method included in Excel's solver function was used to minimize the *RSS* value, which was generalized from the linear programming problems used in the simplified gradient to nonlinear programming problems. When the minimum value of *RSS* was obtained by the gradient method, the local minimum value that was not the true value may be derived, so that the calculations were repeated and resulted in the minimum *RSS*.

$$F(x_i) = \left(f_1(x_i) + f_2(x_i) + f_3(x_i) + f_4(x_i) + f_5(x_i)\right) \quad \textbf{(Eq. S2)}$$

$$RSS = \sum_i \left(y_i - F(x_i)\right)^2 \quad \textbf{(Eq. S3)}$$

The area of the measured spectra and the spectrum of each component obtained by fitting were calculated using the trapezoidal formula. Then, the area of the spectrum of each component were divided by the total area of the measured spectra to determine the proportions (%).

Figure S1

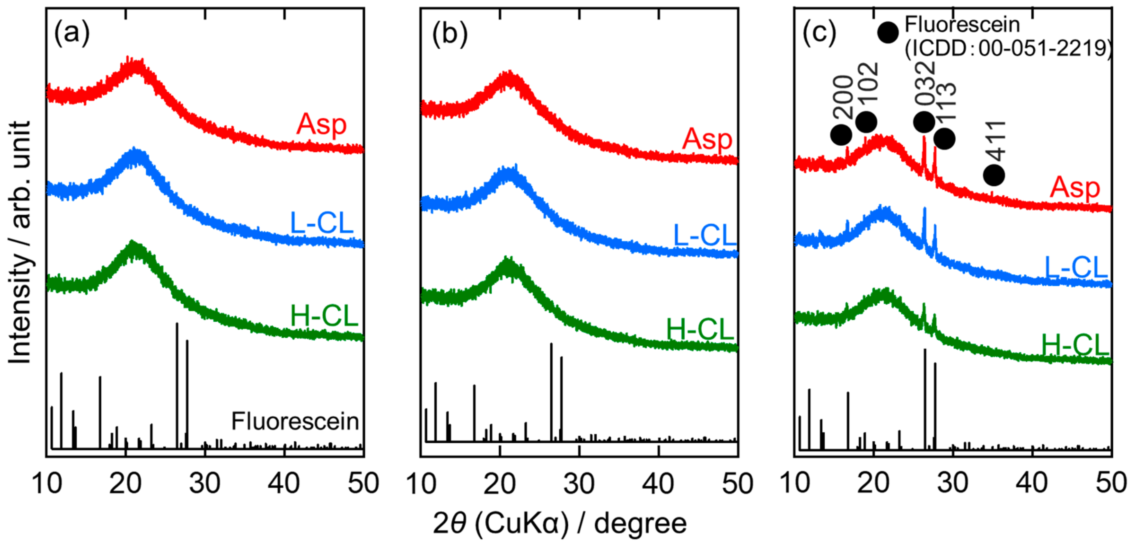

Figure S1. XRD patterns of the FS^- immobilized-ASP washed with (a) EtOH, (b) IPA, and (c) ultrapure water.

Figure S2

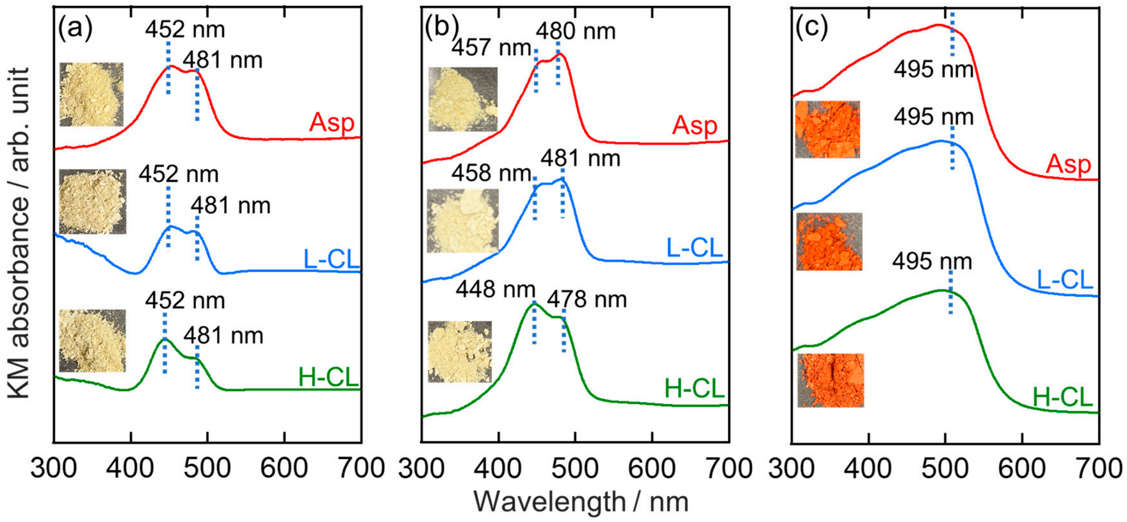

Figure S2. UV-Vis diffuse reflectance absorption spectra of the FS^{-}-immobilized ASP washed with (a) EtOH, (b) IPA, and (c) ultrapure water (Inset: photographs of the powder states).

Figure S3

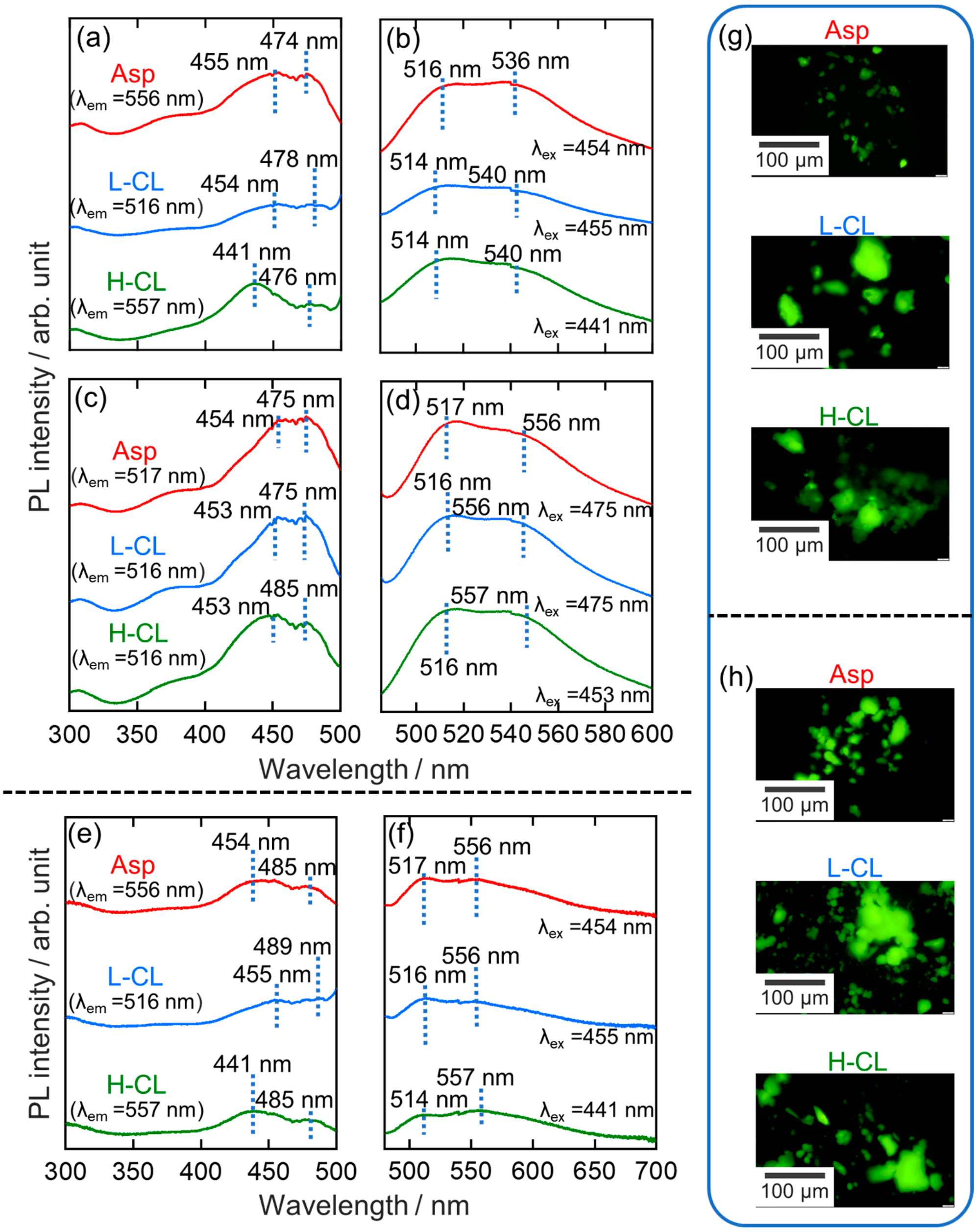

Figure S3. Excitation and luminescence spectra ((a, b) EtOH, (c, d) IPA, and (e, f) ultrapure water washed) of the FS^--immobilized ASP. The monitored luminescence and excitation wavelengths for the spectra are represented by λ_{em} and λ_{ex}. Fluorescent microscope images of the FS^--immobilized ASP washed with the (g) EtOH and (h) IPA (λ_{ex} = 465−495 nm, λ_{em} = 515−555 nm, exposure time = 100 ms).

Figure S4

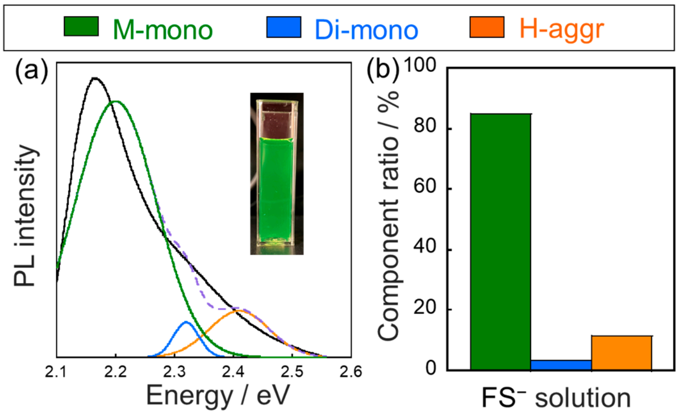

Figure S4. (a) Luminescence spectral separation result for four aggregate states of the FS^- solution (phosphate buffer (1 mM), pH=7.4), and the detailed assignments are shown in **Table S1**. (b) Component ratio for four-aggregate states was calculated by the separated spectra.

Figure S5

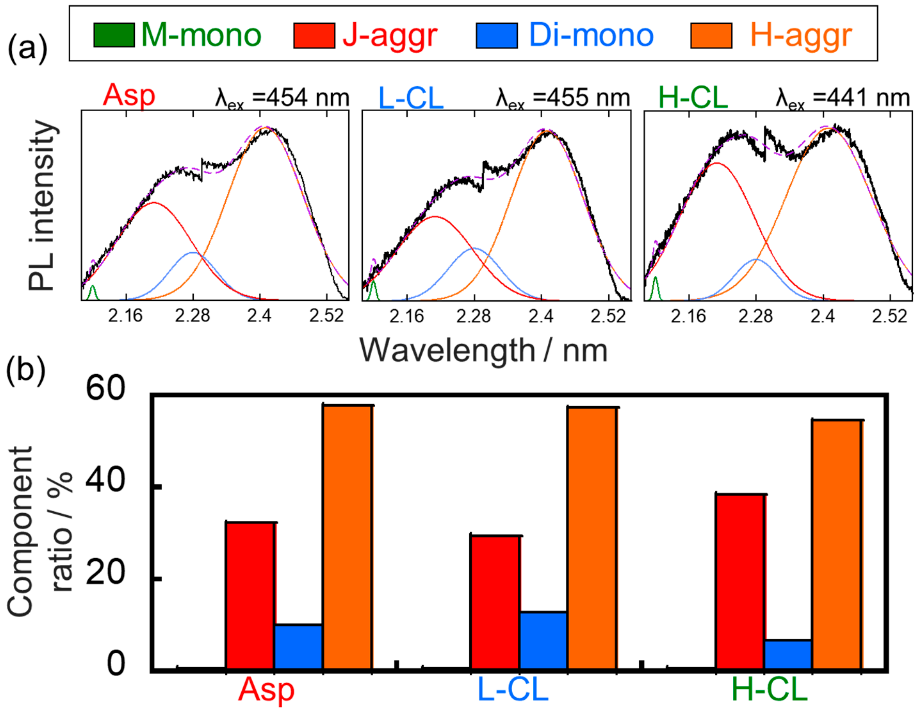

Figure S5. (a) Luminescence spectral separation results for four aggregate states of the FS^- -immobilized ASP washed with the ultrapure water. The detailed assignments were shown in **Table S1**. (b) Component ratio for four-aggregate states was calculated by the separated luminescence spectra.

Figure S6

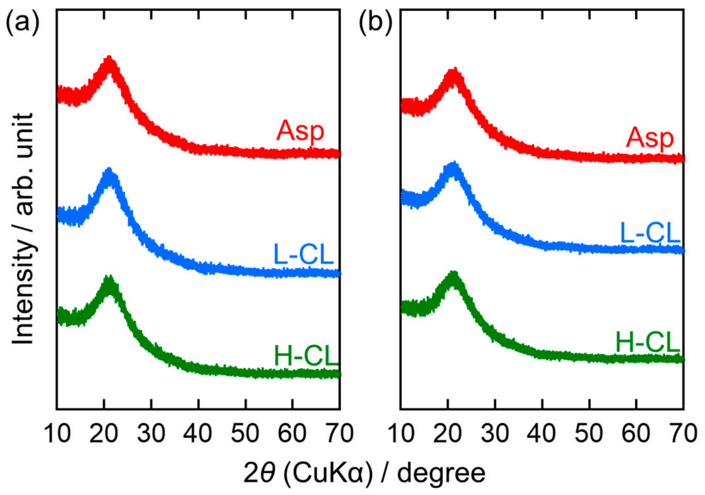

Figure S6. XRD patterns of the MB^{+}-immobilized ASP washed with the (a) EtOH and (b) IPA.

Figure S7

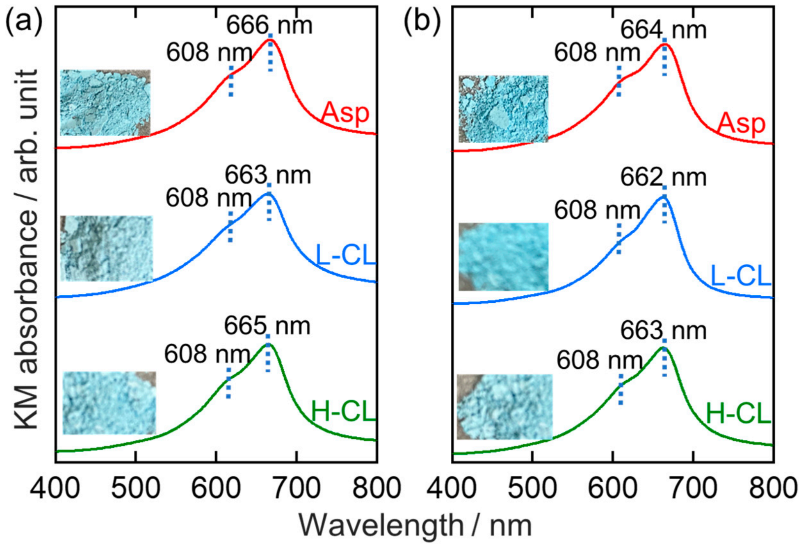

Figure S7. UV-Vis diffuse reflectance absorption spectra of the MB^{+} immobilized ASP washed with (a) EtOH and (b) IPA (Inset: photographs of the powder states).

Figure S8

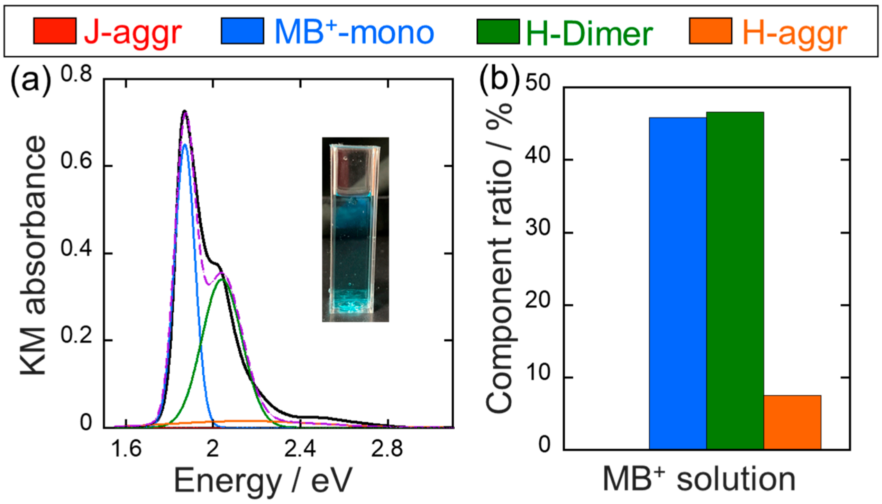

Figure S8. (a) UV-Vis absorption spectral separation result for four aggregate states of the MB^+ solution (phosphate buffer (1 mM), pH=7.4). The detailed assignments were shown in **Table Ap-1**. (b) Component ratio for the four-aggregate states.
